# Phonon Interference Effects in GaAs-GaP Superlattice Nanowires

**DOI:** 10.1021/acsnano.5c10312

**Published:** 2025-12-08

**Authors:** Chaitanya Arya, Johannes Trautvetter, Jose M. Sojo-Gordillo, Yashpreet Kaur, Valentina Zannier, Arianna Nigro, Fabio Beltram, Tommaso Albrigi, Alicia Ruiz-Caridad, Lucia Sorba, Riccardo Rurali, Ilaria Zardo

**Affiliations:** 1 Departement Physik, Universität Basel, Basel 4056, Switzerland; 2 NEST, Istituto Nanoscienze-CNR and Scuola Normale Superiore, Pisa I-56127, Italy; 3 Institut de Ciencia de Materials de Barcelona, ICMAB, CSIC, Campus UAB, Bellaterra 08193, Spain

**Keywords:** nanowires, superlattice, thermal conductivity, phonon coherence, boundary scattering, phonon interference

## Abstract

Fine-tuning the functional properties of nanomaterials is crucial for technological applications. Superlattices, characterized by periodic repetitions of two or more materials in different dimensions, have emerged as promising areas of investigation. We present a study of the phonon interference effect on thermal transport in GaAs-GaP superlattice nanowires with sharp interfaces between the GaAs and GaP layers, as confirmed by high-resolution transmission electron microscopy. We performed thermal conductivity measurements using the so-called thermal bridge method on superlattice nanowires with a period varying from 4.8 to 23.3 nm. The measurements showed a minimum of the thermal conductivity as a function of superlattice period up to room temperature that we interpreted as an indication of the crossover from coherent to incoherent thermal transport. This effect is not destroyed by the surface boundary or by phonon–phonon scattering, as the crossover trend is also observed at room temperature. Our results were corroborated by both *ab initio* lattice dynamics and semiclassical nonequilibrium molecular dynamics calculations. These findings provide insights into the wave-like and particle-like transport of phonons in superlattice nanowires and demonstrate the potential for engineering thermal properties through precise control of the superlattice structure.

## Graphical abstract

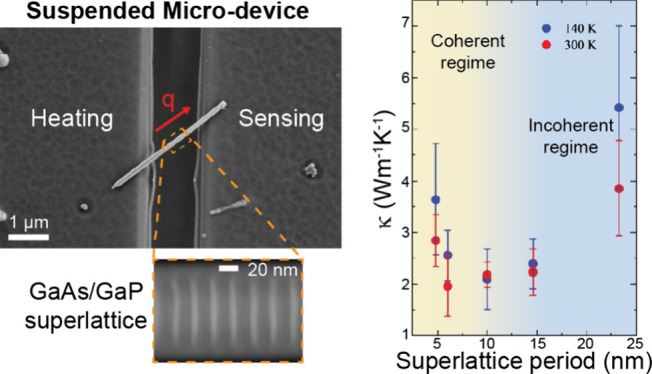

## Introduction

In the latest decades, continuous efforts have been carried out in order to enhance or suppress thermal transport in materials for numerous technological applications.^1,2^ To this end, it is crucial to understand and control phonons by different methods. Namely, phonons are the quanta of lattice vibrations and are the main carriers of sound and heat in insulators and semiconductors. Specifically, low frequency (∼kHz) phonons are responsible for sound transmission, while high frequency (∼THz) phonons are responsible for heat transport. Therefore, one way to control and engineer heat transport consists of manipulating high frequency phonons by modifying the materials at the nanoscale, i.e., the length scale comparable to the phonon mean free path.^3^ While a well-established route to achieve this goal is nanostructuring,^4^ more elaborated yet promising approaches consist of the combined use of nanostructuring and heterostructuring to control the heat transport by means of interference effects that can be achieved in the coherent phonon transport regime.^5–7^

Nanowires (NWs) are nanostructures characterized by a high aspect ratio, featuring a rod-like shape with diameters of the order of few to tens or hundreds of nanometers and lengths of the order of micrometers. They are promising candidates for studying phonon interference effects in different transport regimes because they offer unique possibilities, both in terms of geometry, e.g., with axial^8^ and radial^9^ heterostructures, and in terms of materials, as they release the strain in the radial direction, thus enabling the defect-free combination of lattice matched^10^ or mismatched materials.^11,12^ Furthermore, they allow the growth of high-quality junctions^13^ and have characteristic length scales that nowadays can be controlled with high precision.^14^ It is also technologically relevant that NWs offer advantages over, e.g., two-dimensional systems, as they can readily be integrated into nanoscale device architectures such as thermoelectric generators,^15^ sensors,^16^ and quantum devices,^17^ where size, scalability, and directional heat transport are critical.

Particularly relevant for material engineering are superlattices (SLs), i.e., lattices made by different materials periodically alternated, that can be used to investigate the behavior of phonons scattered by interfaces. Typically, phonons scattered from single interfaces lose their phase information, leading to diffusive thermal transport, whereas a periodic repetition of interfaces can lead to constructive interference, resulting in coherent phonon transport.^18–22^ For these phenomena to occur, it is crucial for the interfaces to be as clean as possible, i.e., defect-free and sharp. On perfectly smooth interfaces, phonons scatter specularly and can interfere constructively with the reflected phonons provided that they are in phase, resulting in altered dispersion relations and in the formation of bandgaps.^2^ The presence of periodically repeated interfaces can further modify the vibrational properties or phonon spectra, as wave interferences can influence the density of states and group velocities of the phonons.^19^

While there is a significant potential for wave interference effects to impact thermal devices, demonstrations of these effects on macroscopic thermal transport quantities are still discussed. SLs provide an ideal platform for studying and understanding coherent phonon effects on macroscopic thermal properties.^23,24^ Depending on the period of the SL and on its relation to the coherence length of phonons, a wave-particle crossover is expected to occur:^25–27^ when the SL period is smaller than the coherence length of the phonons, the wave nature of phonons becomes evident, leading to the appearance of interference effects; on the other hand, when the SL period is larger than the phonon coherence length, phonons are better described as particles that undergo individual and uncorrelated scattering events. The experimental observation of coherent heat conduction was first reported in 2012 by Luckyanova et al.^28^ In that study, the thermal conductivity (κ) of GaAs/AlAs SLs with a varying number of periods was measured using the time-domain thermal reflectance technique in the temperature range from 30 to 300 K. In the coherent regime, the phonon phase information is preserved at the interfaces of the SL. The superposition of Bloch waves leads to the creation of stop bands, effectively modifying the phononic band structure. Consequently, they observed a linear dependence of the thermal conductivity on the total SL thickness over a temperature range of 30 to 150 K, suggesting that phonons can maintain phase coherence across multiple interfaces. On the other hand, in the incoherent regime, phonons are diffusively scattered at each internal interface, causing them to lose their phase information. The interfaces act as independent thermal resistors, leading to an effective thermal conductivity perpendicular to the interfaces that is approximately independent of the number of layers of the SL and that tends to the alloy of the two constituent materials.^29^

Another significant signature of thermal transport across SLs is the presence of a minimum in κ as the interface density (number of interfaces for unit length) varies.^26,30–32^ This minimum is an indication of a transition from particle-like to wave-like transport of phonons, as first proposed by Simkin and Mahan.^26^ The first conclusive experimental evidence of this transition from particle-like (incoherent) to wave-like (coherent) processes was obtained through measurements of lattice thermal conductivity as a function of interface density in epitaxial oxide SLs.^29^ This wave-particle crossover manifests as the existence of a minimum in κ as a function of interface density.^19^

However, the transition from coherent to incoherent behavior has mainly been explored in 2D superlattices, and similar studies in 1D nanostructures are less prevalent. The phonon confinement in two directions might enhance wave interference effects and may allow coherent transport to persist even at higher temperatures. One of the open questions in these systems is the impact of surface boundary scattering on the coherence of phonons and their interference. Yet, 1D systems offer greater potential for heterostructuring and the creation of high-quality NW junctions. We have previously demonstrated the tunability of the phononic spectrum by analyzing the dependence of both acoustic and optical phonon modes on the SL period. As the SL period increases, the number of phonon modes also increases, which can be attributed to the larger number of atoms per unit cell.^33^ In this work, we investigated the phonon interference effects in GaAs-GaP SL NWs by measuring the thermal conductivity using the thermal bridge method.

## Results and Discussion

We investigated SL NWs. Specifically, the NWs are composed of four segments, as depicted in the schematic in Figure 1a. The bottom segment consists of 0.5 μm long GaAs, followed by a GaP stem of about 1 μm, an alternating GaAs/GaP superlattice segment, and a GaP segment of about 1 μm at the top. Figure 1b shows the dark field scanning transmission electron microscopy (STEM) image of an exemplary SL segment with a 4.8 nm period and the corresponding energy dispersive X-ray (EDX) map. From the latter, sharp interfaces between GaAs and GaP layers can be seen, and we have quantified the interface abruptness fitting the chemical profiles with an error function. We found a GaAs/GaP interface abruptness of 0.66 ± 0.22 nm and a GaP/GaAs interface abruptness of 0.68 ± 0.25 nm, which is consistent with the previously reported values from the STEM intensity profile obtained across a few SL layers.^34^

**Figure 1:**
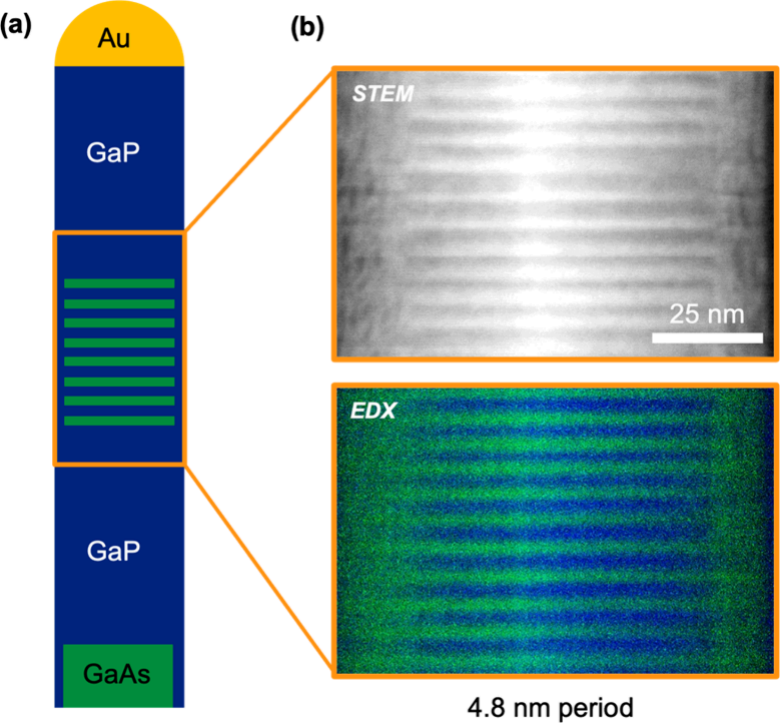
Superlattice nanowire sample. (a) Schematic of a SL NW composed of a GaAs/GaP base segment, a central SL segment, and a top GaP segment. (b) STEM image (top) and corresponding EDX map (bottom) of a SL segment in the middle of a representative NW with 4.8 nm period. The EDX map represents an overlay of the P (blue) and As (green) signals. The scale bar is common for both images.

We used the suspended thermal bridge method (Figure 2) to measure the thermal conductivity (κ) of GaAs-GaP SL NWs for various superlattice periods at bath temperatures from 16 to 350 K. Details on the device fabrication can be found in the Methods section. The NW SL period ranged from 4.8 to 23.3 nm. The SL samples investigated in this work are listed in Table 1 of Supporting Information S1 along with their description.

**Figure 2:**
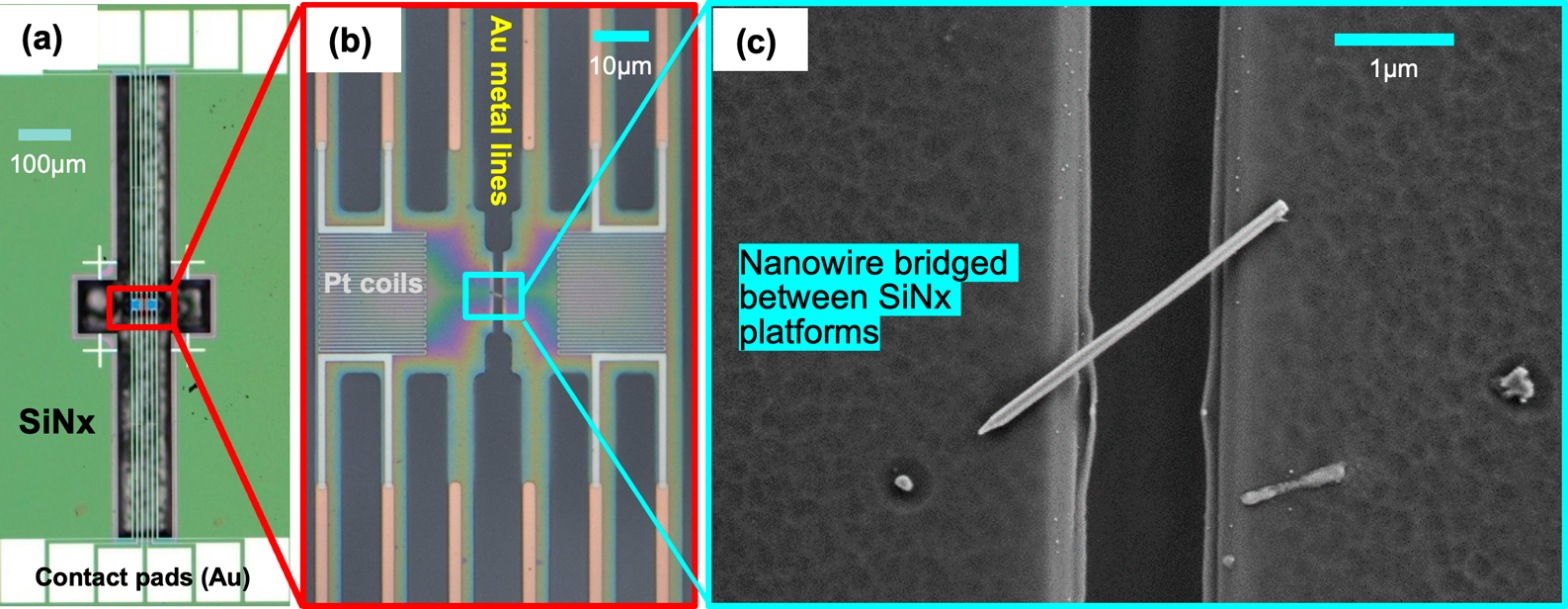
Suspended microdevice for measuring the thermal conductivity of NWs. (a) Optical image of the suspended device. (b) Left and right Pt resistors at the center supported by a long SiN_*x*_ beam with gold metal lines on top. (c) SEM image of a SL NW suspended between the SiN_*x*_ platforms.

The temperature dependent κ results are summarized in Figure 3. Figure 3a shows the measured κ of four NWs with the smallest SL period (4.8 nm) at 300 K, depicting the typical statistical distribution and reproducibility in κ for a given period. The slight variation in conductivity of different NWs can be attributed to the different surface roughnesses, variation in the diameter ranging from 114 to 130 nm (Supporting Information S1), and contact thermal resistances with the platforms.

**Figure 3:**
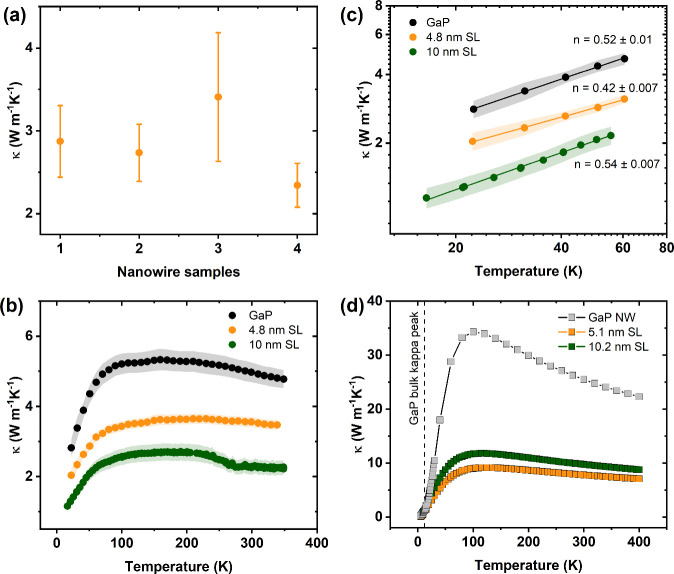
(a) Thermal conductivities of four different NWs featuring a 4.8 nm superlattice period measured at 300 K. (b) Thermal conductivities measured for NWs featuring 4.8 nm (orange) and 10 nm (dark green) superlattice periods and GaP reference NW (black) from 16 to 350 K. The error is represented by the shaded area. (c) Low temperature experimental data plotted on a logarithmic scale. The solid lines are linear fits to the logarithmic data. The error is represented by the shaded area. (d) Computed thermal conductivities of 5.1 and 10.2 nm superlattice period bulk material and a GaP NW with a diameter of 76 nm. The vertical dashed line shows the temperature at which the thermal conductivity of bulk GaP is maximum, i.e., 12 K. In all plots, circle symbols represent experimental data, while square symbols stand for theoretically calculated values.

Further, we perform a temperature dependent measurement of κ for the 4.8 and 10 nm period SL NWs, as well as for a GaP reference NW from 16 to 350 K, shown in Figure 3b. At each bath temperature, experiments were performed for both directions of thermal bias, and the value of the κ was calculated averaging the results of the two measurements. The temperature dependence found is characteristic of NWs, i.e., it increases with *T* ^*n*^ at low temperatures due to the growing phonon population, but eventually, additional scattering mechanisms strongly limit the magnitude.^30,35^ Hence, it resembles closely that of Si NWs with diameters between 40 and 120 nm as reported by Li and co-workers.^36^ Likewise, in our studied NWs, the maxima in κ are shifted to higher temperatures as the additional contribution of boundary scattering masks Umklapp scattering. Moreover, the κ of SL NW peaks at higher temperatures—173 K for the 10 nm period and 218 K for the 4.8 nm period—compared to the GaP reference nanowire, which peaks at 161 K. This is markedly different from the case of bulk GaAs, where κ exhibits a sharp maximum around 20 K followed by a characteristic Umklapp scattering-driven *T* ^–1^ dependence.^37,38^ A similar behavior is found in bulk GaP with κ peaking at 30 K.^39^ Since our three studied NWs share comparable diameters, we expect a similar level of size confinement. Therefore, we attribute the observed shift in the maxima of κ to higher temperatures to the presence of periodic interfaces in the SL structures, which would further suppress high-frequency phonons beyond the effects of boundary scattering.

Figure 3c shows the low temperature range of the measured κ on the logarithmic scale from 15 to 70 K. We roughly see a *T* ^1/2^ dependency for the three studied sample NWs. This trend is remarkably different, e.g., from the case of bulk GaAs, where κ exhibits a *T* ^3^ dependence.^37,38^ This suggests that, at these low temperatures, the boundary scattering produced by the finite diameter of the NWs dominates the phonon propagation as opposed to the contribution of the SL, which would require phonon coherency lengths (roughly the same order of magnitude as the phonon mean free path) proportional to the SL periods to have a significant impact. Therefore, since we do not observe a clear relationship between the trends in κ of pure GaP NW and the SL NWs studied, we cannot conclude that the effect of the SL is significant for *T* < 70 K.

In order to further understand these measurements, we plotted in Figure 3d the computed κ of 5.1 and 10.2 nm GaAs/GaP SL bulk material and, additionally, the κ of a pure GaP NW with a diameter of 76 nm. There results were based on *ab initio* calculations, where we used the VASP code^40^ to perform density-functional theory (DFT) calculations of the harmonic and anharmonic force constants and then solve the Boltzmann Transport Equation (BTE). In the case of SLs, we used the method implemented in almaBTE^41^ that allows bypassing the explicit calculation of the phonon scattering rates in the superlattice unit cell—which would be unfeasible at the *ab initio* level—and rather relies on the phonon properties of the constituent materials, which were carefully determined in advance.^42^ The reliability of this methodology is witnessed by the very good agreement with experimental results on Si/Ge^43^ and InAs/GaAs SLs.^44^ As coherence is destroyed outside the SL computational cell used for the solution of the BTE, in order to avoid artifacts, we used a supercell made of 60 repetitions of the wurtzite (WZ) unit cell for all periods (convergence tests with 120 repetitions were satisfactorily conducted). This length allows to accommodate almost exactly all the periods displayed in the plot (full details on the calculations are provided in the Supporting Information S5). Here, we notice that the absolute values obtained for κ are higher, as additional effects such as sample-device contact resistances or boundary scattering in the case of SLs could not be taken into account. Nevertheless, in all cases, we also observe a shift of the maxima of the thermal conductivity from bulk values (12 K for GaP) to higher temperatures (125 K for 5.1 and 115 K for 10.2 nm SL period), in accordance with the experimental observations. We also notice how the SL structure itself seems also to play a role in shifting the temperature at which κ reaches a maximum, particularly for short periods and around the coherent-incoherent crossover, which occurs for SL periods of ca. 8 nm as exposed by SL period dependent thermal conductivity measurements (see Supporting Information Figure 5, where we plot the theoretical κ­(*T*) for different periods of a bulk SL).

Subsequently, we systematically measured κ as a function of superlattice periods at 300 and 140 K; see Figure 4a. With the exception of the longest period (23.3 nm), for each SL period, we have measured at least two wires under the two directions of thermal bias, and on each wire, the measurements were repeated 5 times to verify the reproducibility of the measurements. In Figure 4a, we plot the average of the measured thermal conductivities for each period as a function of the SL period. Noteworthy, the measured thermal conductivity is the result of the combined contributions of the SL in series with one of the pure GaP segments (see Figure 1) and of the contribution of contact resistances.

**Figure 4:**
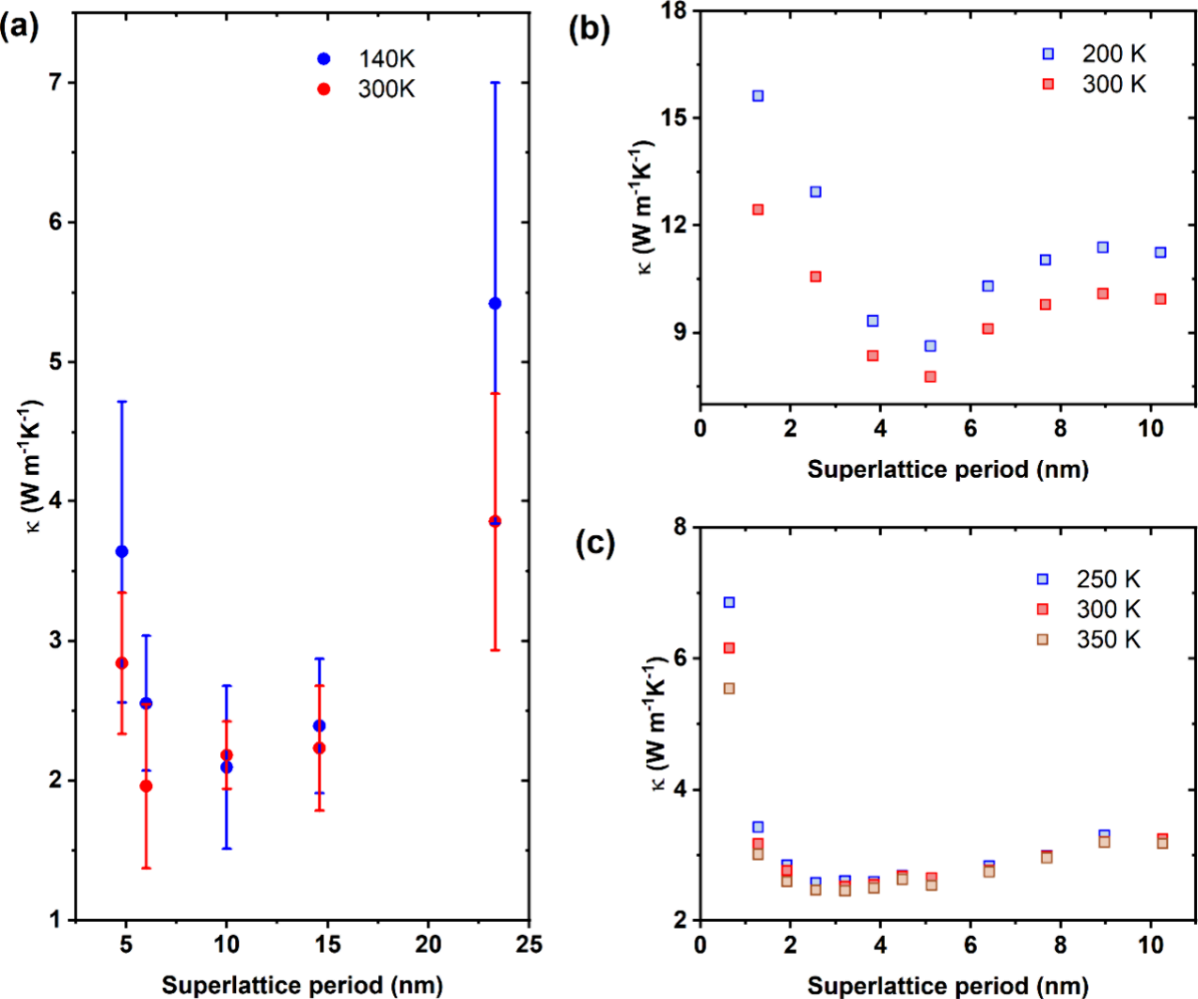
(a) Average thermal conductivity of superlattice nanowires as a function of superlattice period at 300 (red) and 140 K (blue). The errors correspond to the standard deviations of multiple measurements. (b) Computed thermal conductivity as a function of period in GaAs/GaP SLs obtained from DFT/BTE and (c) NEMD calculations. The DFT/BTE calculations are carried out in a bulk system, while in NEMD, we considered a NW of 5 nm diameter. In all plots, circle symbols represent experimental data, while square ones stand for theoretically calculated values.

Starting from large periods, with decreasing SL period length, the thermal conductivity value decreases. This decrease in κ can be attributed to an increase in phonon scattering due to the increased number of interfaces. For this range of SL periods (>8 nm), the phonon coherence length is smaller than the period length; thus, reducing the latter simply creates a further number of interfaces (per unit length), causing higher phonon scattering and thus yielding a lower overall κ. Around SL period sizes of ∼8 nm, the conductivity value reaches a minimum, and then, it starts to increase again with decreasing period length. This occurrence of a minimum in the thermal conductivity for SL NWs as a function of decreasing SL period is a signature of the transition from an incoherent to the coherent phonon transport regime. In the coherent regime, i.e., for SL periods smaller than the coherence length, phonon phase information is preserved at the interfaces of the SLs and interference between phonons and their reflections occurs, leading to a modification of the phonon dispersion with the formation of mini-bands and possibly with the opening of forbidden energy gaps. In particular, the number of interfaces determines the number of mini-bands formed, which is directly related to the average phonon group velocity. Therefore, an increase in the number of interfaces yields an enhancement of the thermal conductivity, as observed for the NWs with a period of 4.8 nm in Figure 4a. Comparing the data set obtained for the two base temperatures, the trend is similar, though the minimum of κ is more pronounced at low temperature, and the minimum seems to shift toward longer SL periods, as expected from the increase in phonon mean free path (MFP) at low temperature and in agreement with previous findings.^29^

Our results are particularly promising as they demonstrate a coherent phonon interference effect in 1D systems, despite their high surface-to-volume ratio. Namely, in order to maintain phase coherence, phonons must scatter specularly at the surface boundaries of nanostructures. Typically, the phonon-boundary scattering is diffusive and suppresses the phonon MFP. Our results instead indicate the preservation of phonon coherence up to room temperature. We could speculate that the presence of the shell around the SL visible in Figure 1b,c (see also Methods and Supporting Information S1) fosters specular scattering and thus helps preserve coherency. In particular, it is worth noticing that the GaP shell around the SL provides a space layer between the SL itself and the oxidized GaP outer layer. The SL/GaP interface has a better quality than the one between GaP and amorphous oxide and could, therefore, provide a higher rate of specular scattering.

Furthermore, these results shine light on the possibility of obtaining coherent phonons in highly strained systems. Indeed, GaAs and GaP have a 3.7% lattice mismatch. The ability to achieve coherent phonon transport in such nanostructures highlights the potential for engineering thermal properties at the nanoscale, even in systems in which strain effects are more pronounced.

Subsequently, we corroborated our experimental results against two different computational frameworks. First, in Figure 4b, we report our results of κ as a function of the period of a bulk GaAs/GaP SL based again on DFT calculations as previously described. As can be seen, we obtain a minimum in κ­(L) around 5 nm at both temperatures considered. Similar to the experiments, for a small period, we observe a sharp increase in κ, the fingerprint that the transport regime has become coherent and interfaces do not act anymore as individual, independent barriers. The increase in the thermal conductivity in the diffusive regime depends on the atomistic structure of the interfaces and thus on the associated thermal boundary resistance (TBR). In the Supporting Information S5, we also show data for idealized, atomically flat interfaces, where phonon scattering is entirely dominated by Umklapp processes, due to the low value of the TBR, and κ­(*L*) for large *L*’s is flat (the contribution of the TBR of each interface is so small that the difference between having, e.g., 4 or 5 interfaces is negligible). The comparison between the results of Figure 4b and Figure S5 highlights the important role of the value of the TBR and of the underlying model used to account for it. It is worth noting that we compute the TBR within a phenomenological, though usually accurate, model (i.e., the diffuse mismatch model (DMM); see Supporting Information S5). This is most likely the reason for some discrepancy with the experimental results (e.g., κ exhibits small changes with increasing period from 5 to 10 nm in Figure 4a, while it increases by almost a factor of 2 in Figure 4b).

Finally, we have also performed computational experiments based on nonequilibrium molecular dynamics (NEMD) with the LAMMPS code^45^ and a bond-order potential^46^ (full details on the calculations are provided in Supporting Information S5). These calculations are complementary to the *ab initio* calculations discussed above. While they cannot be expected to have a predictive power, being based on an empirical interatomic potential not especially designed to reproduce thermal transport properties, they allow one to account explicitly for the NW geometry and do not need to rely on any phenomenological model for the TBR. Our results for a GaAs/GaP NW SL with a diameter of 5 nm and a total length of 100 nm and for three different base temperature conditions are shown in Figure 4c, where we plot the thermal conductivity as a function of SL period. Notice that, due to computational limitations, the diameters of the NWs used in the NEMD simulations are considerably smaller than those used in the experiments and thus these results can only be taken qualitatively. We find a minimum in κ that falls at a shorter period length with respect to the *ab initio* results. This is a qualitative indication that the NW geometry and a more realistic description of the TBR do not hinder *per se* the appearance of a minimum in κ (*L*) and thus the onset of a coherent transport regime at short periods.

For a fair comparison between the experimental and theoretical data sets, it is worth highlighting that while experiments are performed on NWs, where boundary scattering, surface roughness, and finite cross-sectional effects play a major role, the DFT/BTE calculations were performed for bulk superlattices with infinite cross-section and sharp though interfaces. On the other hand, in NEMD calculations, the diameters differ significantly, and boundary scattering plays a very important role in NWs. Therefore, the comparison between experiments and theoretical calculations can be qualitative. Furthermore, the different *y*-scales for the three different panels of Figure 4 should be noted.

## Conclusions

We performed experiments and numerical simulations to investigate the thermal conductivity of GaAs-GaP SL NWs with varying superlattice periods, exploring the transition from incoherent to coherent phonon transport as the superlattice period decreases. Experimental results show the temperature dependence of the highly suppressed thermal conductivity of SL nanowires due to the combined effect of the boundary and TBR scatterings. Remarkably, we observed a decrease in thermal conductivity with decreasing period length, reaching a minimum at around 8 nm, indicative of an increased phonon scattering at interfaces that later crossovers toward a coherent transport for shorter SL periods, where the thermal conductivity was found to increase again. *Ab initio* calculations support these findings, showing a minimum thermal conductivity at approximately 5 nm. Computational experiments using nonequilibrium molecular dynamics, where the nanowire geometry is explicitly accounted for, vouch for the generality of these observations. We have demonstrated the preservation of the coherence of phonons up to room temperature in NWs, despite the importance of surfaces in these nanostructures, possibly arising from specular boundary scattering. Our findings display an interesting way to tune thermal properties by carefully designing a material system such as a superlattice nanowire. This additional degree of control, combined with the inherent confinement of phonons in 1D systems, can readily yield enhanced thermal performance of microdevices such as thermoelectric generators or sensors, where the scalability and directionality of heat transport are key features.

## Methods

GaAs-GaP SL NWs with different periodicities and uniform thickness used for this study were grown using Au-assisted chemical beam epitaxy (CBE) on a GaAs (111)B substrate with the same conditions detailed in prior work.^34^ High resolution TEM and STEM images for the whole set of investigated nanowires can be found in Supporting Information S1, along with the EDX maps of NWs with 4.8 and 23.3 nm SL periods. For all investigated samples, the SL NW’s core diameter ranges from 30 to 50 nm and the shell around the NW has 20 nm thickness and it mainly consists of GaP. We performed thermal transport experiments on SL with periodicities ranging from 4.8 to 23.3 nm. Most of the SLs were composed of 100 repetitions, while samples with 14.6 and 23.3 nm periods had 30 repetitions without a top GaP segment.

The thermal conductivity of SL NWs was measured using the well-established suspended thermal bridge method proposed by Shi et al.^47^ To ensure thermal isolation, we fabricated a microdevice consisting of gold deposited on 0.5 mm long suspended SiN_*x*_ beams. These SiN_*x*_ beams supported two suspended platforms in the center, which contained platinum resistors acting as a heater or temperature sensor, while the beams had gold patterned lines, which led to contact pads for electrical connections. In the device fabrication process, the design of gold lines and platinum resistors on SiN_*x*_ was accomplished using optical and electron beam lithography techniques, respectively, while gold and platinum metals were deposited using electron beam evaporation followed by lift-off in acetone. Subsequently, the device was prepared for suspension. This involved a series of etching steps, both dry and wet, to create the required suspended structure by selectively removing the materials. Finally, the SiN_*x*_ membranes were carefully cut to create a gap between the two platforms. This was done using a focused ion beam (FIB) to ensure a high control in the gap dimensions (∼1 μm). This is crucial because the SL NWs used in our study are not very long and require small gaps between bridges. Supporting Information S2 provides more details on the specific steps and parameters involved in the device fabrication process.

The suspended devices were calibrated in a probe station (Janis ST-500) under vacuum (10^–5^ to 10^–6^ mbar). Experiments at low temperature (below RT) were performed with liquid helium cooling. Electrical measurement of the resistors featured by the suspended devices was carried out using a source-meter unit (Keithley 4200A-SCS Parameter Analyzer) in a four-wire configuration. Contact with the chip was achieved using multiprobe tips. Temperature dependent measurements were instead carried out using the closed-cycle cryostat by Advanced Research Systems (ARS DE200) in a variable temperature environment ranging from 10 to 355 K, with temperature stability within ±0.1 K during operation. All thermal measurements, i.e., calibration and data acquisition, were carried out under high-vacuum conditions (∼10^–5^ mbar) to suppress heat dissipation to the surrounding air.

Prior to the thermal conductivity measurements, these resistors required calibration. During this process, the change in resistance of the platinum resistors is measured with respect to the base temperature variations and with respect to the power dissipated in each meander. This data allows for the calculation of the coefficient of thermal resistance d*R*/d*T* as well as the beam conductance *G*_B_ = (d*R*/d*P*)/(d*R*/d*T*) = dΔ*T*/d*P* of both platforms. Both parameters are required for subsequent measurements (more details in Supporting Information S3). After the calibration process, GaAs-GaP NWs were transferred from the original substrate with vertical arrays of NWs onto the suspended devices between the two platforms using a hydraulically actuated micromanipulator. To measure the thermal conductance of the NW, a controlled temperature difference was created between the two platforms. One platform was heated, while the change in temperature on the second platform, due to thermal transport through the NW, was measured as a function of the applied heating power. The temperature of the platforms was obtained by measuring the resistances using a four-point probe technique, which serves as a reliable indicator of temperature variations thanks to the linear temperature coefficient of resistance of the platinum lines of the resistor. The heat flux across the NW was then calculated by measuring the power dissipated by the resistors. As depicted in Figure 2c, the NW bridges both platforms, forming a thermal pathway. When a bias current is applied through the heater platform, heat is generated, and some of this heat is transferred through the NW to the sensor platform. As a result, the temperature of the sensing resistor increases and, thus, its resistance. This parameter is then simultaneously recorded as a function of the applied power on the heater side. Hence, the NW thermal conductance is calculated as (details in Supporting Information S4)^47,48^

$$G_{N}=G_{B,S}\frac{d\Delta T_{S}}{dP}{\left(\frac{G_{B,H}}{G_{B,S}}\cdot \frac{d\Delta T_{H}}{dP}-\frac{d\Delta T_{S}}{dP}\right)}^{-1}$$

where $\frac{d\Delta T_{H}}{dP}$ and $\frac{d\Delta T_{S}}{dP}$ are the heater and sensor temperature increase rates, respectively, as a function of the dissipated heater power and *G*_B,H_ and *G*_B,H_ are the heater and sensor beam conductances, respectively, calculated as $\frac{d\Delta T_{H}}{dP}$ when the device is measured without a NW sample bridging both platforms. Finally, the thermal conductivity of the NW is calculated as $\kappa =\frac{4G_{N}L}{\pi D^{2}}$ , where *L* is the NW suspended length and *D* is the diameter of the wire.

## Supplementary Material



## Data Availability

The data that support the findings of this study are openly available in ZENODO at https://doi.org/10.5281/zenodo.15719471.

## Notes

Superlattice nanowire samples; Device fabrication process (Fabrication steps of the thermal bridge device); Device characterization (Measurement steps for device characterization); Nanowire conductance measurements; Details of the theoretical calculations (Lattice dynamics calculations, Molecular dynamics calculations) (PDF)
