# Supplementary material for: Phonon Interference Effects in GaAs-GaP Superlattice Nanowires
Source: ACS Nano. 2025 Dec 8;19(50):42013–21. doi: 10.1021/acsnano.5c10312 (PMC12752693; doi:10.1021/acsnano.5c10312)
Supplement: Supplementary file 1 [file nn5c10312_si_001.pdf]

# Supporting information

## Phonon interference effects in GaAs-GaP superlattice nanowires

*Chaitanya Arya[1], Johannes Trautvetter[1], Jose M. Sojo-Gordillo[1], Yashpreet Kaur[1], Valentina Zannier[2], Arianna Nigro[1], Fabio Beltram[2], Tommaso Albrigi[3], Alicia Ruiz-Caridad[1], Lucia Sorba[2], Riccardo Rurali[3] and Ilaria Zardo[1*]*

[1]Departement Physik, Universität Basel, 4056 Basel, Switzerland
E-mail: Ilaria.zardo@unibas.ch

[2]NEST, Istituto Nanoscienze-CNR and Scuola Normale Superiore, I-56127 Pisa, Italy

[3]Institut de Ciència de Materials de Barcelona, ICMAB−CSIC, Campus UAB, 08193 Bellaterra, Spain

## S1: Superlattice nanowire samples

Table 1: Summary table of all samples used in this study.

| **GaAs thickness (nm)** | **GaP thickness (nm)** | **SL period (nm)** | **Repetition** | **SL segment length (μm)** | **Nanowire diameter (nm)** | **SL position** |
|---|---|---|---|---|---|---|
| 2.0 | 2.8 | 4.8 | 100 | 0.48 | 114 - 130 | center |
| 3 | 3 | 6 | 100 | 0.6 | 120 - 140 | center |
| 4.2 | 5.8 | 10 | 100 | 1 | 130 | center |
| 5 | 5 | 10 | 100 | 1 | 110 - 130 | center |
| 6.5 | 8.1 | 14.6 | 30 | 0.438 | 100 | tip |
| 10.7 | 12.6 | 23.3 | 30 | 0.699 | 90 | tip |

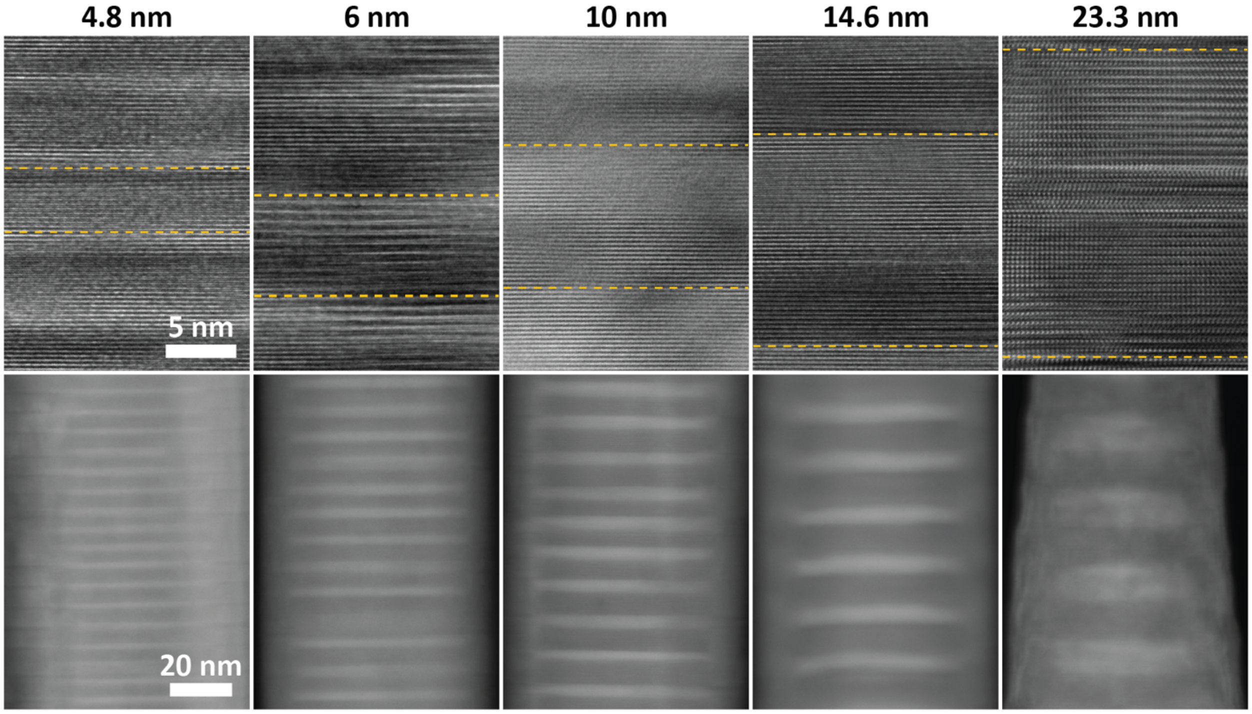


Figure S1. Top row: High Resolution Transmission Electron Microscopy (HRTEM) images of representative NWs from each SL design (periods are indicated above the panels). The dashed lines highlight the position of the interface between the different material layers. The scale bar is common to all images of the top row. Bottom row: Dark Field Scanning Transmission Electron Microscopy (STEM) images of the corresponding NWs from the top row. The scale bar is common to all images of the bottom row.

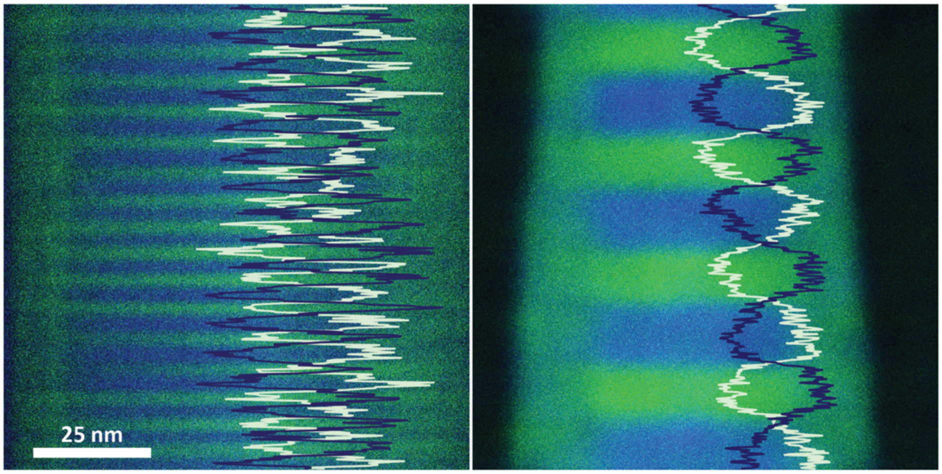


Figure S2. Energy dispersive X-ray (EDX) maps of representative NWs with 4.8 nm (left panel) and 23.3 nm (right panel) SL period. The maps represent an overlay of the P (blue) and As (green) signals. The line profiles represent the normalized intensities of the P and As signals. The scale bar is common for both maps. We have extracted the interface steepness fitting the chemical profiles with an error function. For the nanowire with 4.8 nm SL period, we found a GaAs/GaP interface steepness of 0.66 ± 0.22 nm and a GaP/GaAs: 0.68 ± 0.25 nm. For the nanowire with 23.3 nm SL period, we found a GaAs/GaP interface steepness of 2.10 ± 0.26 nm and a GaP/GaAs: 1.76 ± 0.25 nm.

## S2: Device fabrication process

To accurately measure the thermal conductivity of a single nanowire (NW), a sensitive platform is required to measure the temperature gradient across the NW and calculate the heat flux through it. One crucial requirement is that the platform must be isolated from the surrounding environment to prevent heat dissipation. Therefore, the experiments are typically conducted in a vacuum environment to minimize heat loss to the surroundings. In order to calculate the NW's thermal conductivity, one end of the wire is heated while the temperature at this end is precisely controlled and measured by recording the change in resistance. Simultaneously, the temperature at the other NW's end is also measured. Fourier's law is then used to compute the thermal conductivity of the NW. By knowing the heat flux and the temperature gradient across the NW, the thermal conductivity can be determined [1].

Figure S3 shows a scanning electron microscope (SEM) image of the suspended device fabricated on a silicon substrate. The device consists of two suspended platforms made of silicon nitride ($SiN_x$). These platforms are equipped with metal lines and are separated by a small gap. The central region of the device consists of platinum resistors, while gold is deposited on the lines. The platinum resistors have a higher resistance compared to the gold lines, resulting in the majority of the power being dissipated in the resistors. The gold pads, which are not suspended, serve as heat sinks. To perform the electrical measurements, multiprobe tips are employed to contact the gold pads. These tips enable the application of current and the measurement of voltage, allowing for precise control and monitoring of the electrical properties of the device.

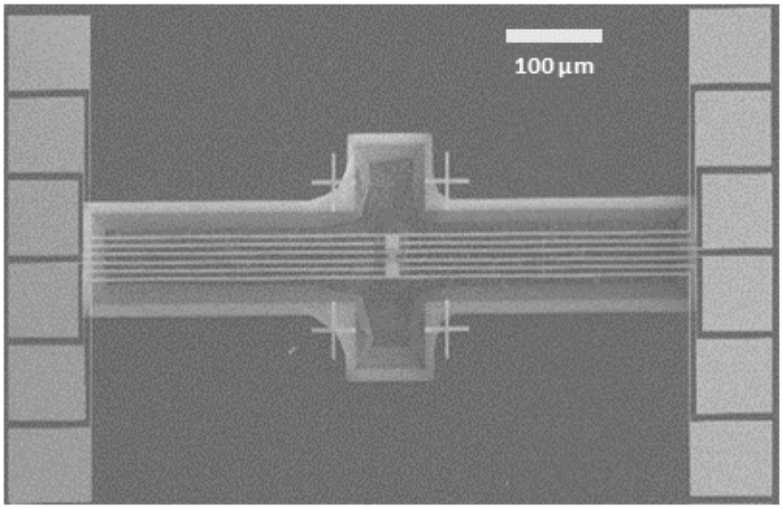


Figure S3. SEM image of a suspended microdevice showing the contact pads connected to suspended platforms at the center through suspended beams.

**Fabrication of thermal bridge device**

The fabrication of the suspended device involves several stages to accommodate the different feature sizes present in the device (see Figure S4). The first stage utilizes a laser writer, while the second stage employs electron beam lithography for patterning the smallest features with high precision. Subsequently, steps of dry and wet etching are carried out to suspend the microstructures.

**$SiN_x$ deposition** – The fabrication process begins by depositing a 525 nm thick layer of $SiN_x$ onto a two-inch undoped <110> silicon wafer. The deposition is performed using a plasma-enhanced chemical vapor deposition (PECVD) technique with a PlasmaPro PP80 PECVD system by Oxford instruments, which yield good-quality films [2]. During the deposition process, achieving a high-quality film with low strain is crucial for the successful suspension of the device at the final stage. To accomplish this, high and low frequency plasma conditions are applied for 16 and 4 second cycles, respectively, at supplied power of 20 W to alternatively deposit film with compressive and tensile strain to achieve a $SiN_x$ film with very low stress [3].

**Laser lithography** – After the $SiN_x$ deposition, the next step in the fabrication process involves the creation of the contact pads and lines using optical lithography. The wafer is cleaned with acetone and IPA followed by prebaking for 5 minutes. To achieve a clean lift-off process, two different positive resists are utilized to form a bi-layer to achieve an undercut profile. First, LOR3A resist is spin-coated onto the wafer at 4000 rpm for 45 seconds, followed by a soft baking step at 190 ºC for 2 minutes. Next, S1805 resist is spin-coated at 4500 rpm for 40 seconds and soft baked at 125 ºC for 2 minutes. Then the wafer is aligned in the laser writer, and the desired patterns are exposed onto the resist layer. Subsequently, the wafer is developed using a 2% tetramethylammonium hydroxide (TMAH) MF-319 solution for 1 minute, which selectively removes the exposed resist. To ensure complete removal of the developing solution and residues, the wafer is rinsed several times in deionized (DI) water.

**Metal deposition (Au)** – To create the metal contact pads and lines, a titanium (Ti) layer with a thickness of 3 nm, followed by a thin layer of gold (Au) with a thickness of 27 nm, is deposited using Electron Beam Evaporation. Before the deposition process, the sample undergoes a cleaning step using oxygen plasma for 10 seconds to remove any residual resist from the exposed regions. The deposition of Ti serves as an adhesion layer to the $SiN_x$ substrate. For lift-off, the sample is immersed in acetone in a bath set at a temperature of 50 ºC for a duration of 1 hour.

**Electron beam lithography** – First the wafer is cleaned by Oxygen plasma (60W) for 5 minutes to ensure that no resist residual is left after the lift off. In this case, PMMA is spin coated (5000 rpm, 40s) and baked at 180 ºC for 5 minutes. After the exposure, the wafer is developed in solution of 2% MIBK and IPA in a ratio of 1:3 for 1 minute 10 seconds and rinsed in IPA.

**Metal deposition (Pt)** – The resistors are fabricated by depositing a 3 nm layer of Titanium (Ti) followed by a 27 nm layer of Platinum (Pt) using Electron Beam Evaporation. Again, Ti acts as an adhesion layer. The lift-off process is performed in acetone at 50 ºC to for 1 hour followed by rinsing in IPA.

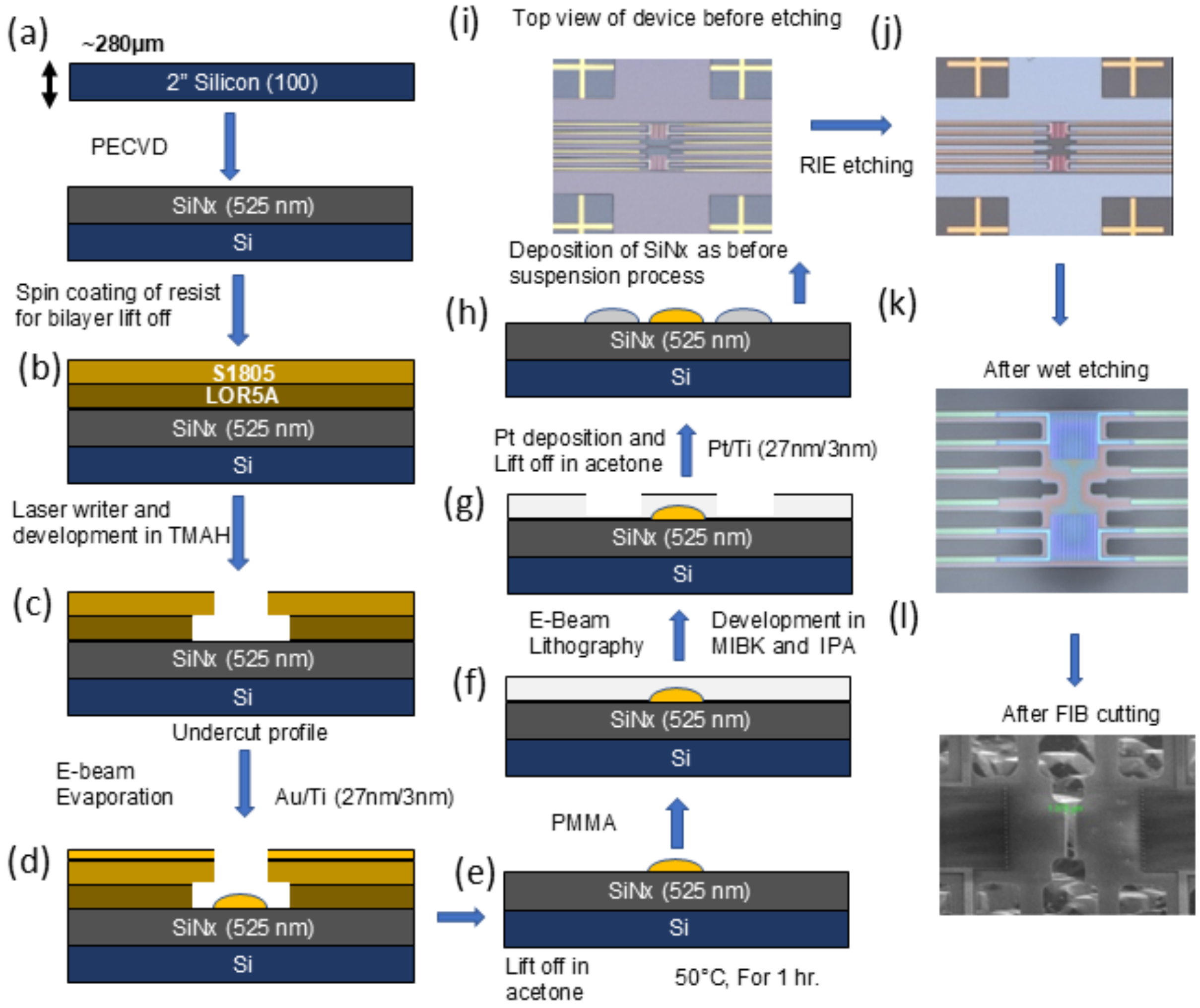


Figure S4. Schematic of fabrication steps for a suspended microdevice. a) CVD $SiN_x$ layer deposition. b-e) Lift-off process for patterning the Au contacts and beams using laser lithography. f-h) Lift-off process for patterning the Pt contacts and beams using e-beam lithography. i) Top view of the device after SiNx deposition to protect the metals during etching process. j) RIE etching of the $SiN_x$. k) Wet etching of the devices to suspend them. l) FIB cutting of the micro-platforms to pattern the desired gap.

**Device Suspension** – To enable proper thermal isolation and create a temperature gradient across the resistors, the device undergoes a suspension process. The regions surrounding the metal lines are selectively etched to remove the substrate material, to ensure that heat generated in the resistors is not dissipated into the substrate. Meanwhile, resist masks are used to protect the metal lines during the etching process. This suspension step is crucial for enabling effective heat transport through the NW and accurate thermal conductivity measurements.

To protect the metal lines during the wet etching process, a 450 nm layer of $SiN_x$ is deposited on the entire wafer using the PECVD. A photoresist layer (S1805) is then spin-coated onto the $SiN_x$ layer and patterned using optical lithography to create an opening in the mask for the etching region. The exposed sample is developed in a 2% TMAH (MF-319) solution for 1 minute. Reactive ion etching is used to selectively etch away the $SiN_x$ layer underneath the exposed regions, utilizing a mixture of $CHF_3$ and $O_2$ plasma. The process is carried out at 60 W applied power, 60 mTorr pressure, and gas flows of 80/8 sccm for $CHF_3/O_2$, resulting in an etch rate of approximately 35 nm per minute. The thickness of the photoresist is chosen such that it is etched away at the similar time as the $SiN_x$, ensuring complete removal of the resist and $SiN_x$ from the exposed areas. For silicon etching, a wet etching process is carried out using TMAH at 80 ºC for 7 hours, followed by KOH etching at 50 ºC for 10 minutes [4]. This etching process removes the silicon substrate in the areas where the $SiN_x$ layer has been etched away by dry etching process, suspending the beams and platforms with metal resistors and lines.

**Focused Ion Beam** – In the final step of the fabrication process, the two platforms of the device are separated using a focused ion beam (FIB). Using the FIB, a desirable gap of approximately 1 µm is created between the two suspended platforms.

## S3: Device characterization

Before measuring the thermal conductivity of the NWs, it is crucial to determine the thermal conductance of the suspended beams ($G_B$). The beam conductance $G_B$ represents the thermal conductance from the suspended membrane to the surrounding environment at a reference temperature $T_0$. This conductance accounts for the heat transfer occurring between the suspended membrane and the environment. The thermal conductance $G_B$ is calculated using Fourier's law (Equation 1.1). $\dot{Q}$ is the rate of heat transfer and $\Delta T$ is the temperature difference.

$$\dot{Q} = G_B \cdot \Delta T \tag{1.1}$$

The heat is generated through Joule heating in the platinum resistors. The power P dissipated in the resistors equals the heat transfer rate, $\dot{Q}$ = P, and equation 1.1 can written as

$$P = G_B \cdot \Delta T \qquad (1.2)$$

with P = R · $I^2$, where P represents the power, R is the resistance of the resistors, and I is the current flowing through the resistors and depicted in Figure S5 (a). Assuming a linear relationship between the power dissipated in the resistors and the temperature rise in the suspended membrane, Equation 1.2 can be written as

$$G_B = \frac{P}{\Delta T} = \frac{dP}{dT} = \frac{dR/dT}{dR/dP}\,. \qquad (1.3)$$

The power dissipated in the resistor leads to a temperature rise in the suspended membrane resulting in a temperature difference, ΔT, between the center platform and the ends of the beams (contact pads). By measuring the resistance change in the resistors, where the majority of power is dissipated (platinum resistance is much higher than gold lines), the temperature rise in the suspended membrane can be determined. Figure 3 (a) shows the schematic of the measurement system for the one platform of the suspended device. Where $T_0$ is temperature of the substrate, T is temperature of the center platform, P is the power dissipated in the Pt resistor. The current is applied using multi probe tips and the voltage is measured to calculate the resistance of the Pt resistors.

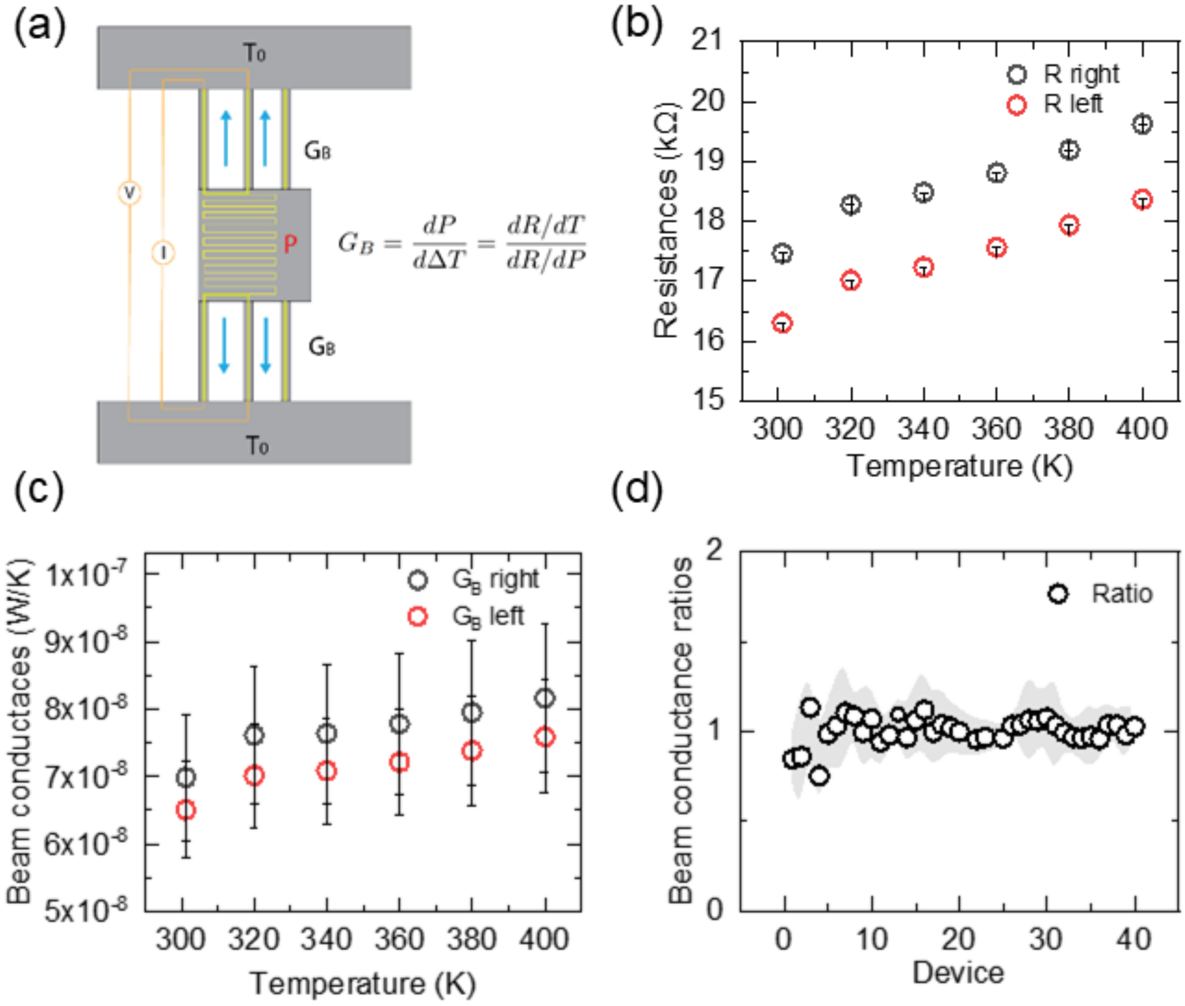


Figure S5. (a) Schematic showing the method for beam conductance measurements. (b) Resistance of platinum resistors with respect to temperature. (c) Beam conductance of left and right beams with temperature, and (d) beam conductance ratio of all the measured devices showing the device asymmetry.

**Measurement Steps for Device Characterization:**

Temperature Stabilization: The sample stage is first heated to a specific base temperature to ensure a stable and controlled thermal environment. This step allows the device to reach thermal equilibrium before measurements begin, minimizing any transient thermal effects.

Resistance Measurements: Small currents, ranging from -450 nA to 450 nA, are applied to the device. At each current level, the 4-point resistance is measured, ensuring precise readings by

eliminating contact resistance and the Au line contributions. The measured resistance values are then plotted as a function of temperature. From this data, the temperature derivative of resistance, dR/dT, is calculated (Figure S5 (b)).

Gradient Application: To extract the derivative dR/dP, a gradient is applied to the device. This additional measurement step helps quantify how resistance varies with an increasing power.

Using the obtained values of dR/dT and dR/dP, the beam conductance $G_B$ is calculated based on Equation 1.3. Figure S5 (c) illustrates the measured conductance for both the left and right beams as a function of temperature, beam conductance increases with rising temperature, but the ratio between the left and right beam conductance remains constant. Additionally, Figure S5 (d) presents the beam conductance ratio for all measured devices, with the x-axis representing the device number. This illustrates the variation in the asymmetry factor (the conductance ratio of left and right beam) of the fabricated devices [5].

## S4: Nanowire conductance measurements

To assess the thermal conductance of the NW, we start by passing current through the beams to one of the resistors, inducing a temperature increase $\Delta T_H$ on the heater side of the platform due to Joule heating. As heat passes through the NW, a small $\Delta T_S$ is observed on the sensor platform. The schematic in Figure S6 depicts the components of the thermal bridge and their respective heat fluxes. We estimate the heat flow through the NW by measuring the power dissipated in the resistors via a 4-point measurement.

Once we have determined the total power heating up the platforms, we can apply the principle of energy conservation to the entire system to estimate the conductance of the beams as follows:

$$P_H + P_S = G_{B,H}\Delta T_H + G_{B,S}\Delta T_S \tag{2.1}$$

where $P_H$ and $P_S$ represent the power dissipated in the heater and sensor resistors, respectively; $G_{B,H}$ and $G_{B,S}$ denote the beam conductance on the heater and sensor sides, respectively; $\Delta T_H$ and $\Delta T_S$ represent the temperature rise with respect to the base temperature $T_0$ on the heater and sensor sides, respectively.

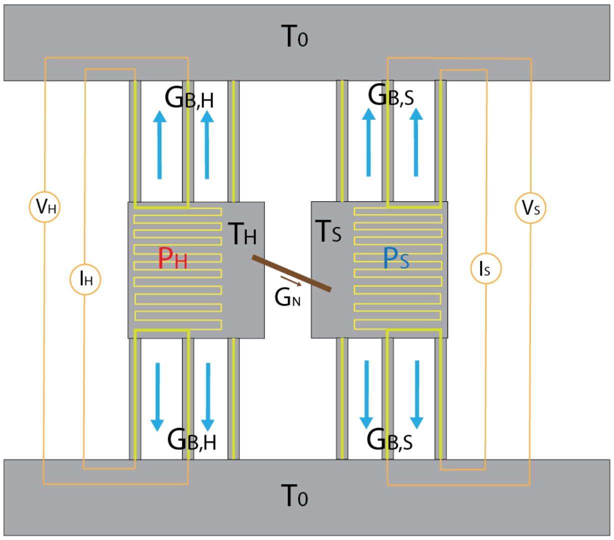


Figure S6. Schematic illustration of suspended microdevice used for measuring the thermal conductivity of NWs. The direction of heat flow is indicated by arrows.

From the energy balance on the sensor side, we obtain:

$$G_N(\Delta T_H - \Delta T_S) + P_S = G_{B,S}\Delta T_S \, . \tag{2.2}$$

From equation (2.1):

$$G_{B,S} = (P_H + P_S) \cdot \left(\frac{G_{B,H}}{G_{B,S}}\Delta T_H + \Delta T_S\right)^{-1} . \tag{2.3}$$

In order to use slopes from the measurements of power and temperature, we assume that power and temperature follow a linear relationship. Thus, we can write

$$\frac{d\Delta T_x}{dP} = \Delta T_x \, / (P_H + P_S) \tag{2.4}$$

and rewrite Equation (2.3) as:

$$G_{B,S} = \left(\frac{G_{B,H}}{G_{B,S}} . \frac{\mathrm{d}\Delta \mathrm{T_H}}{\mathrm{dP}} + \frac{\mathrm{d}\Delta \mathrm{T_S}}{\mathrm{dP}}\right)^{-1} . \tag{2.5}$$

Using the Equation 2.2, the NW conductance $G_N$ can be given as

$$G_N = G_{B,S}\frac{d\Delta T_S}{dP}\left(\frac{G_{B,H}}{G_{B,S}} \cdot \frac{d\Delta T_H}{dP} - \frac{d\Delta T_S}{dP}\right)^{-1} \tag{2.6}$$

where $\frac{d\Delta T_H}{dP}$ and $\frac{d\Delta T_S}{dP}$ are the slopes of temperature rise on the heater and sensor side, respectively, as a function of total power dissipated, and $G_{B,H}$ and $G_{B,S}$ are the heater and sensor beam conductance, respectively. $\frac{G_{B,H}}{G_{B,S}}$ is defined the asymmetry factor of the device beams' conductance. The thermal conductivity of the measured NW can be extracted using the following Equation

$$k = \frac{4G_N L}{\pi D^2}$$

where L and D, are the suspended length and diameter of the NW [1,6].

## S5: Details of the theoretical calculations

**Lattice dynamics calculations.** We perform density-functional theory (DFT) calculations with the VASP [7,8] code, projector-augmented wave (PAW) potentials [9,10], and the local density approximation (LDA) for the exchange correlation energy. The unit cells of bulk wurtzite GaAs and GaP were optimized until strict convergence criteria for stress ($3\times10^{-3}$ GPa) and forces ($5\times10^{-4}$ eV/Å), sampling the Brillouin zone with a 16×16×12 Γ-centered mesh. Harmonic and third-order anharmonic interatomic force constants (IFCs), were computed in 4×4×3 supercells using the phonopy [11] and the thirdorder.py code [12]. Interactions beyond fourth nearest neighbors were neglected in the case of anharmonic IFCs. The linearized phonon Boltzmann Transport Equation (BTE) was solved with the methods implemented in the almaBTE code [13], where superlattices are modelled as a periodic perturbation upon a reference virtual crystal [14]. We consider rather sharp, but gradual interfaces, where the chemical composition switch from GaAs to GaP within two-unit cells (the composition if 100% GaAs in cell *i*, 75% GaAs and 25% GaP in cell *i+1*, 25% GaAs and 75% GaP in cell *i+2*, and finally 100% GaP in cell *i+3*). We also considered idealized, atomically flat interfaces, where phonon scattering is entirely dominated by Umklapp processes, due to the low value of the thermal boundary resistance (TBR). In this case κ(L) flattens, rather than increasing, as L increases (see Figure S7), because of the low contribution of the TBR of each interface (having e.g. 4 or 5 interfaces results in a negligible difference).

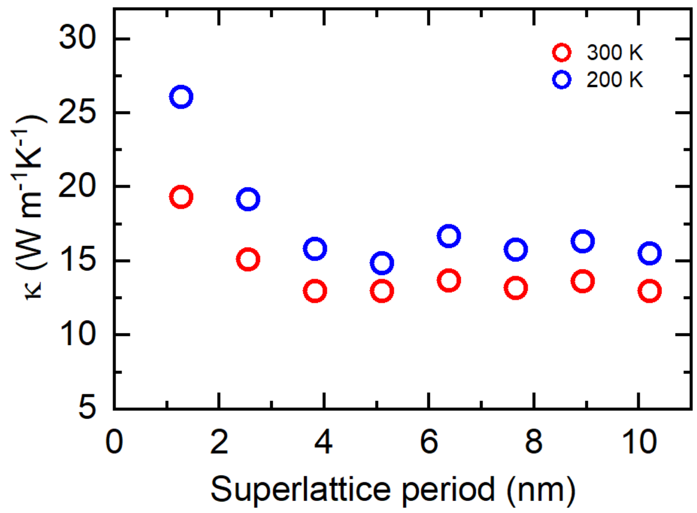


Figure S7. Computed thermal conductivity as function of the period in GaAs/GaP SLs with idealized flat interfaces.

In Figure S8 we plot the dependence of the thermal conductivity as a function of temperature, κ(T), for all the SL investigated. As it can be seen, for the shortest period, when transport is fully coherent, κ(T) resembles that of a homogeneous material, with a rater sharp peak at low temperature. As the period increases and coherence is progressively lost, the peak shifts toward higher temperature and becomes blunter. This trend continues until the period reaches 5.1 nm, which is where we observe the coherent-incoherent crossover and is then reversed, with the maximum of κ(T) shifting back to lower temperature and tending to become sharper (this trend would become more evident for longer periods, which we cannot afford computationally). Notice that for temperatures $\lesssim 50$ K these results have to be taken only qualitatively, because transport is dominated by phonons of increasingly long wavelength and the **q-point** meshes required to achieve strict convergence criteria cannot be handled efficiently.

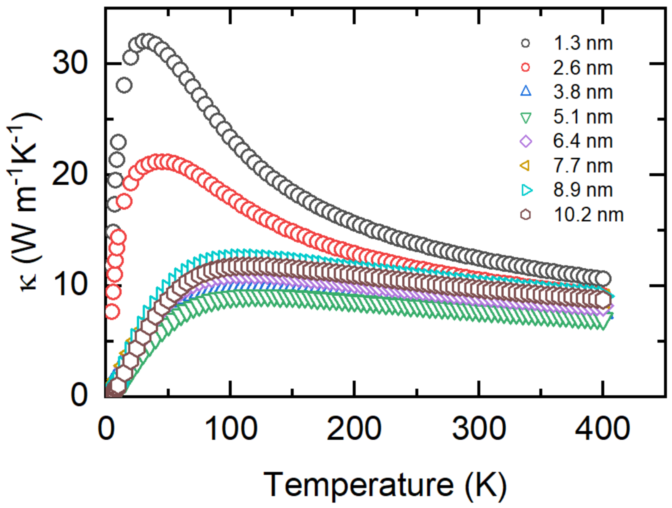


Figure S8. Computed thermal conductivity as function of temperature in GaAs/GaP SLs of different periods, L.

**Molecular dynamics calculations.** We performed nonequilibrium molecular dynamics (NEMD) simulations with the LAMMPS code [15,16] by imposing a temperature bias of 170 K, with the hot and cold reservoirs set at 385 K and 215 K, respectively. The timestep was set to 0.7 fs, and the total simulation time was 7 ns. After an initial structural relaxation, the temperatures of the simulation cell ends are controlled by rescaling the velocities of the atoms therein, while the central region is left free to evolve without constraints; full details of the simulation protocol can be found e.g. in Ref. [17]. We account for the atomic interactions by mean of the bond-order classical potential due to Powell *et al* [18]. The mixed interactions between atoms belonging to neighboring GaP and GaAs segments were derived using the empirical approach of Tersoff in the case of SiC and SiGe [19]. The NW structure was generated by stacking the appropriate supercell until reaching the target length of approximately 100 nm. Subsequently, atoms outside the hexagonal cross-section with an edge length of 2.5 nm were removed, resulting in a NW with a final diameter of 5 nm. The NWs studies are oriented along the <111> crystallographic axis and have a length of approximately 100 nm.

# References

[1] Shi L, Li D, Yu C, et al. Measuring Thermal and Thermoelectric Properties of One-Dimensional Nanostructures Using a Microfabricated Device. Journal of Heat Transfer. 2003;125(5):881–888.

[2] Yi K, Liu D, Chen X, et al. Plasma-Enhanced Chemical Vapor Deposition of Two-Dimensional Materials for Applications. Acc Chem Res. 2021;54(4):1011–1022.

[3] Ftouni H, Blanc C, Tainoff D, et al. Thermal conductivity of silicon nitride membranes is not sensitive to stress. Phys Rev B. 2015;92(12):125439.

[4] Vassili Karanassios, George Mew. Anisotropic Wet Chemical Etching of Si for chemical analysis applications. Sensors and Materials. 9(7):395–416.

[5] Kaur Y, Tachikawa S, Swinkels MY, et al. Thermal Rectification in Telescopic Nanowires: Impact of Thermal Boundary Resistance. ACS Appl Mater Interfaces. 2025;

[6] Swinkels MY, Van Delft MR, Oliveira DS, et al. Diameter dependence of the thermal conductivity of InAs nanowires. Nanotechnology. 2015;26(38):385401.

[7] Kresse G, Hafner J. *Ab initio* molecular dynamics for liquid metals. Phys Rev B. 1993;47(1):558–561.

[8] Kresse G, Furthmüller J. Efficient iterative schemes for *ab initio* total-energy calculations using a plane-wave basis set. Phys Rev B. 1996;54(16):11169–11186.

[9] Blöchl PE. Projector augmented-wave method. Phys Rev B. 1994;50(24):17953–17979.

[10] Kresse G, Joubert D. From ultrasoft pseudopotentials to the projector augmented-wave method. Phys Rev B. 1999;59(3):1758–1775.

[11] Togo A, Chaput L, Tadano T, et al. Implementation strategies in phonopy and phono3py. J Phys: Condens Matter. 2023;35(35):353001.

[12] Li W, Carrete J, A. Katcho N, et al. ShengBTE: A solver of the Boltzmann transport equation for phonons. Computer Physics Communications. 2014;185(6):1747–1758.

[13] Carrete J, Vermeersch B, Katre A, et al. almaBTE : A solver of the space–time dependent Boltzmann transport equation for phonons in structured materials. Computer Physics Communications. 2017;220:351–362.

[14] Chen P, Katcho NA, Feser JP, et al. Role of Surface-Segregation-Driven Intermixing on the Thermal Transport through Planar Si / Ge Superlattices. Phys Rev Lett. 2013;111(11):115901.

[15] Fast Parallel Algorithms for Short-Range Molecular Dynamics. Journal of Computational Physics. 1995;117(1):1–19.

[16] Thompson AP, Aktulga HM, Berger R, et al. LAMMPS - a flexible simulation tool for particle-based materials modeling at the atomic, meso, and continuum scales. Computer Physics Communications. 2022;271:108171.

[17] López-Güell K, Forrer N, Cartoixà X, et al. Phonon Transport in GaAs and InAs Twinning Superlattices. J Phys Chem C. 2022;126(39):16851–16858.

[18] Powell D, Migliorato MA, Cullis AG. Optimized Tersoff potential parameters for tetrahedrally bonded III-V semiconductors. Phys Rev B. 2007;75(11):115202.

[19] Tersoff J. Modeling solid-state chemistry: Interatomic potentials for multicomponent systems. Phys Rev B. 1989;39(8):5566–5568.
